# Effect of paternal overweight or obesity on IVF treatment outcomes and the possible mechanisms involved

**DOI:** 10.1038/srep29787

**Published:** 2016-07-14

**Authors:** Qingling Yang, Feifei Zhao, Linli Hu, Rui Bai, Nan Zhang, Guidong Yao, Yingpu Sun

**Affiliations:** 1 Reproductive Medical Center, First Affiliated Hospital of Zhengzhou University, China

## Abstract

Leukocyte telomere lengths (LTLs) are shorter in obese compared with normal weight people. However, it is not known whether sperm telomere length (STL) is related to obesity. The aim of the study was to evaluate the impact of men’s body mass index (BMI) on STL, embryo quality, and clinical outcomes in couples undergoing IVF. In total, 651 couples were recruited, including 345 men with a normal BMI and 306 men with an overweight BMI (normal BMI group: 20–25 kg/m^2^; overweight BMI group: >28 kg/m^2^). We found that couples with male’s BMI over 28 kg/m^2^ exhibited a significantly lower fertilization rate, good-quality embryo rate and clinical pregnancy rate compared to their normal BMI counterparts. The mean STL in the overweight BMI group was also significantly shorter than that of the normal BMI group. The results also showed that individuals with higher BMI had higher ROS (Reactive oxygen species) content and sperm DNA fragmentation rate when compared with normal BMI individuals. Mitochondrial activity was also lower in the overweight BMI group than in the normal BMI group. This is the first report to find that STL is shorter in overweight/obese men, which may account for their poorer treatment outcomes in IVF cycles.

Overweight and obesity are ongoing worldwide epidemics, and their effects on reproductive health have been widely studied in women. Overweight or obese women experience longer times to pregnancy and reduced probabilities of conception relative to their normal weight counterparts^1^,^2^. Moreover, they are at an increased risk of early and recurrent miscarriage^3^. These women generally have reduced oocyte quality, lower developmental potential, and poor clinical outcomes when they undergo assisted reproduction treatment^4^,^5^,^6^. In contrast, few studies have focused on the impact of men’s BMI on assisted reproductive technology (ART) outcomes. In particular, the impact of the male’s BMI on embryonic development during IVF has been rarely studied. Numerous studies have examined the effects of overweight and obesity on semen parameters (e.g., sperm motility, morphology and count) with conflicting results. Several studies have shown a negative association between overweight BMI and standard semen parameters^7^,^8^,^9^, whereas other studies have shown the reverse^10^,^11^. Previous studies have also investigated the associations between BMI, testosterone and sex hormone serum levels, and some found that increased BMI was consistently associated with reduced levels of serum sex-hormone-binding globulin, inhibin B, T (testosterone), and FSH (follicle stimulating hormone)^12^,^13^. However, other studies did not observe these changes^8^,^10^.

Telomeres are highly repeated hexameric (TTAGGG) nucleotide sequences at the ends of chromosomes^14^. In association with bound proteins, telomeres protect the chromosome ends from being recognized as double-strand breaks requiring repair. One study found that the shortening of long telomeres in sperm can help sustain the critical telomere length required for normal embryonic cleavage post-fertilization^15^. Another study showed that wild-type oocytes fertilized by sperm with short telomeres from later generations of telomerase-null mice (TR^−/−^) led to increased embryo fragmentation, low embryo quality, and apoptosis^16^. Our most recent study also demonstrated that sperm telomere length (STL) is positively associated with embryo quality in IVF^17^.

Obesity is often accompanied by oxidative stress and inflammation^18^. Oxidative stress is an important contributing factor to telomere attrition^19^. Several studies have found significant negative associations between BMI and the telomere length of leukocytes in peripheral blood^20^,^21^,^22^,^23^. A recent study found that BMI was positively correlated with reactive oxygen species (ROS) production in semen^24^. However, little is known about the effects of overweight and obesity on STL or the role of these relationships in IVF treatment outcomes. The present study aimed to evaluate the effects of male BMI on fertilization rate, early embryonic development quality, and clinical pregnancy rate in couples undergoing IVF. We found that fertilization rates, high-quality embryo rates and clinical pregnancy rates were lower among couples with men whose BMIs were over 28 kg/m^2^ than with men whose BMIs were between 20 and 25 kg/m^2^. In addition, we assessed the mean STL for each patient, and the results showed that this mean value was significantly shorter in the overweight BMI group than in the normal BMI group. We also found that sperm mitochondrial activity was lower but that the sperm DNA fragmentation rate and ROS content in semen were higher in the overweight BMI group than the normal BMI group.

## Results

### Basic patient characteristics and IVF cycle outcomes

**Figure 1:**
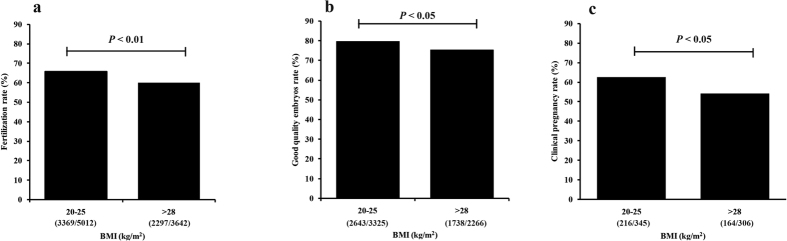
Between-group comparison of fertilization, embryonic development and clinical pregnancy rate (the raw data were shown in the figures). (**a**) Comparison of fertilization rates between couples with men with normal BMIs (20–25 kg/m^2^; N = 345) and overweight BMIs (>28 kg/m^2^; N = 306) (chi-square test). (**b**) Comparison of high-quality embryo rate on day 3 between couples with men with normal BMIs (N = 345) and those with men with overweight BMIs (N = 306, chi-square test). (**c**) Comparison of the clinical pregnancy rate between couples with men with normal BMIs (N = 345) and those with men with overweight BMIs (N = 306, chi-square test).

We enrolled 651 patients including 306 patients with an overweight BMI and 345 age-matched patients with a normal BMI undergoing IVF for the first time. The basic patient characteristics are shown in Table 1. The data are presented as the means ± standard deviations. Male’s age, female’s age, female BMI, female basal FSH level, Gn (gonadotropin hormone) duration, Gn dose, oocytes retrieved and embryos transferred did not significantly differ between the two groups. However, the semen parameter analysis showed that although sperm concentration did not exhibit between-group differences, sperm activity (a parameter assessed in semen analysis that consists of the ability of the sperm to swim in a forward direction), semen volume and total sperm count were significantly higher in the normal BMI group than in the overweight BMI group (Table 2). We also compared the IVF cycle outcomes between the two groups and found that the fertilization rate was significantly lower in the overweight BMI group than in the normal BMI group (Fig. 1a). When we compared the high-quality embryo (grade I and grade II) rate between the two groups, we found that it was significantly lower in the overweight BMI group (Fig. 1b). Furthermore, the clinical pregnancy rate was also significantly lower for this group than for the normal BMI group (Fig. 1c). To confirm the relationship between male BMI and pregnancy rate in IVF, we performed a multivariate logistic regression analysis. After adjusting for female BMI, female age, basal FSH level, male age, number of embryos transferred and sperm count, the data showed that males with a BMI >28 kg/m^2^ were associated with lower clinical pregnancy rates (Table 3).

### Shorter relative STLs in overweight/obese patients

**Figure 2:**
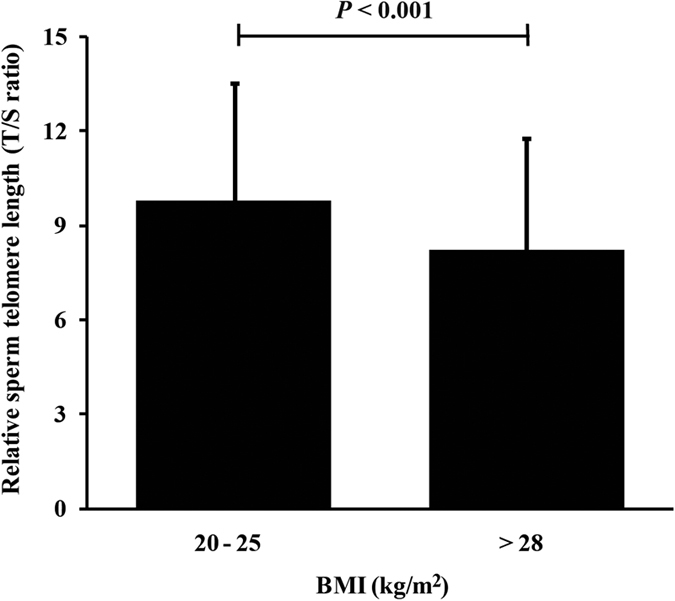
Comparison of relative STL (T/S ratio) in men with normal BMIs (20–25 kg/m^2^; N = 345) and overweight BMIs (>28 kg/m^2^; N = 306) (Student’s *t* test; bars represent standard deviations).

Previous studies, including one from our own lab, have suggested that STL plays an important role in early embryonic development^16^,^17^,^25^. Because obese men usually have shorter LTLs (Leukocyte telomere lengths), we examined the relative STL for each patient. When we compared the STLs between the two groups, we found that the mean relative STL was significantly shorter in the overweight BMI group than the normal BMI group (Fig. 2). Previous studies have also found that STL is positively associated with age.

### Increased oxidative stress, decreased DNA integrity and mitochondrial activity in the sperm of overweight/obese patients

To assess the levels of oxidative stress in the semen collected from the overweight BMI group, we used the NBT method to detect ROS content. We collected semen from another 104 patients, including 54 patients with a BMI >28 kg/m^2^ and 50 patients with normal BMIs. The production of formazan in the serum of men with overweight BMIs was significantly higher than that of those with normal BMIs (Supplementary Figure 1a; *P* < 0.01). This result implies that oxidative stress is prominent in overweight/obese men.

We performed a sperm chromatin dispersion test to determine the DNA fragmentation level for each patient. The results showed that the overweight/obesity group had a higher sperm DNA damage rate than the normal BMI group (Supplementary Figure 1b; *P* < 0.01). Shorter STLs were also found in the overweight BMI group than in the normal BMI group (Supplementary Figure 1c; *P* < 0.05). Those findings suggest that overweight/obese men have decreased DNA integrity. For sperm mitochondrial activity, the percentage of sperm with DAB class II and III was lower (Supplementary Table 1; DAB classes II: *P* < 0.03; DAB classes III: *P* = 0.04) and the percentage of sperm with DAB class IV was higher in overweight (Supplementary Table 1; *P* < 0.01) than in normal BMI men, suggesting that overweight/obese patients have decreased sperm mitochondrial activity.

## Discussion

Infertility affects approximately 15% of couples worldwide, and male factors account for approximately 50% of all cases^26^. However, the mechanisms involved are poorly understood. Over the past two decades, ART has become more popular and has helped many infertile couples have their own children. Previous studies have shown that female obesity has a detrimental influence on fertility and treatment outcomes in IVF; however, relatively few studies have focused on the impact of male overweight/obesity during IVF.

Although several recent studies have explored the effects of male BMI on early embryonic quality and clinical pregnancy rate during IVF, they have reported conflicting results. One study including 344 infertile couples stated that men with a BMI over 25 kg/ m^2^ have a significantly lower chance of having partners achieve clinical pregnancy than men of normal weight; however, day 3 embryo quality was not affected^27^. Another study including 305 infertile couples found that an elevated paternal BMI was associated with a lower clinical pregnancy rate. However, no significant relationship was found with regard to early embryo development^28^. Furthermore, one study including 114 couples found no association between male BMI and IVF fertilization rate, embryo quality on day 3, or pregnancy rate^29^. In the present study, we investigated the effect of overweight/obesity on IVF treatment outcomes and tried to explore the possible mechanisms involved. We studied 651 couples, including 306 couples with a male with an overweight BMI and 345 with a male with a normal BMI. The data showed that sperm activity, semen volume and total sperm count were significantly higher in the normal BMI group than in the overweight BMI group. Although the etiology behind the reduced sperm parameters is not completely clear, several possible factors have been suggested as causes. Some studies found that an elevated BMI can impair spermatogenesis by causing an increase in scrotal temperature^30^,^31^, thus leading to impaired semen quality^32^. Other studies also reported that obesity is associated with increased oxidative stress^33^,^34^, an ROS content most likely higher than the usual metabolic rates required to maintain normal biological processes and an increased level of stress in the local testicular environment. Redundant ROS can lead to sperm DNA damage and affect normal sperm function and motility by disturbing mitochondrial genomes^35^.

In the present study, we also found that couples with a male BMI >28 kg/m^2^ had significantly lower fertilization rates, high-quality embryo rates and clinical pregnancy rates compared with their normal weight counterparts. A recent meta-analysis study in which thirty papers were included demonstrated that paternal obesity negatively affects male fertility and assisted reproduction outcomes, as shown by significantly reduced fertility in the general population and reduced rates of live birth from ART, as well as increased rates of nonviable pregnancy^36^. Combined with previous studies^27^,^28^, our results suggest that male adiposity impairs the treatment outcomes of IVF cycles, although the clear causal mechanism remains unclear. Some researchers have tried to explore the mechanisms of paternal obesity on early embryonic development using a mouse model of high fat diet. One study found some physiological changes to the preimplantation embryo, including significant delays in cell cycle progression, decreased blastocyst development and cell number, and increased glycolytic rate^37^. Functional studies examining blastocyst attachment, growth and implantation showed that blastocysts derived from the sperm of obese males displayed significantly reduced outgrowth on fibronectin and delayed fetal development following embryo transfer^38^. All the above results demonstrated that paternal obesity can affect embryonic development. However, it is not very clear which of the changes in sperm due to obesity ultimately affects early embryonic development.

More and more studies are focusing on investigating the role of telomere length and the telomerase system in reproduction. In telomerase-null mice, the fertilization of wild-type oocytes with sperm with short telomeres from late generations resulted in increased embryo fragmentation, low embryo quality, and apoptosis^16^. One study in humans showed that telomere lengths in cumulus cells could predict highly competent and high-quality oocytes^39^. Another study demonstrated that telomerase was a better predictor of pregnancy outcomes following IVF than telomere length^40^, and our recent study also found that STL was positively associated with early embryonic development in IVF cycles^17^. In addition, several studies have reported the effects of overweight/obesity on LTL^20^,^21^,^22^,^23^. We measured the STL of each patient; thus, this study is the first to evaluate how overweight/obesity affects relative telomere length in human sperm. Interestingly, we found that the mean STL was significantly shorter in males with a higher BMI compared with controls.

In the present study, we also investigated the ROS content in the semen of 54 overweight/obese men and 50 normal weight men. The results demonstrated that the former group had higher ROS levels in their semen than the latter group, which is consistent with previous findings showing that BMI is positively correlated with ROS content in sperm^24^. The present study also found that the sperm mitochondrial activity was lower and the DNA fragmentation rate higher among the overweight/obesity group than the normal weight group, which was consistent with a previous study^41^. Although ROS plays an important role in modulating essential sperm function, imbalances in ROS levels could lead to dysfunction of the mitochondria^42^ and DNA damage^43^. One study showed that when a human telomere sequence was inserted into a plasmid, it caused up to 7-fold more oxidation-induced DNA cleavage in the telomere sequence than a control sequence^44^. Another study found that telomere shortening is significantly increased under mild oxidative stress when compared with that observed under normal conditions in fibroblasts^45^. Over-expression of the extracellular superoxide dismutase gene in human fibroblasts decreased the peroxide content, further decreasing the rate of telomere shortening. All the results demonstrated that telomere length was sensitive to oxidative stress. Additionally, some studies found that high-energy intake can enhance oxidative stress within the testicular environment^46^,^47^. Intracellular ROS can also affect telomere function indirectly by their interactions with telomerase, which can lead to a loss of activity of telomerase reverse transcriptase^48^,^49^. Moreover, white adipose tissue can secrete various hormones, including leptin^50^, that increase sperm oxidative metabolism^51^, thus reducing sperm motility by decreasing axonemal protein phosphorylation, as well as by lipid peroxidation^52^. All the results of the present study combined with those of previous studies suggest that shorter TL, lower mitochondrial activity and higher DNA fragments in the sperm of high BMI patients may be due to increased ROS content.

In summary, we found that STL is closely associated with BMI and that higher BMIs are associated with shorter STLs, decreased mitochondrial activity and increased DNA fragmentation, which might be a result of increased ROS content. All of these changes might lead to lower fertilization rates, embryo quality and clinical pregnancy rates for patients undergoing IVF. Taken together, these findings help us partly understand the effects of paternal overweight/obesity on IVF treatment outcomes. We suggest that obesity increases inflammation and oxidative stress, which might cause shortening of sperm telomere length, ultimately leading to poor early embryo quality and reduced pregnancy rates. However, additional studies are required to determine the detailed causal mechanisms involved.

## Experimental Methods

### Patient selection

Our sample consisted of 306 overweight males (>28 kg/m^2^) and 345 age-matched males of normal weight (20–25 kg/m^2^). All patients were undergoing treatment at our reproductive medical center for first fresh IVF cycles. All patients had normal chromosome karyotypes. None of the men had Y chromosome micro-deletions or any evident causes of spermatogenic impairment. The FSH levels of all of the women were below 10 IU/L. Women were excluded when they had damaged ovarian function that was present prior to the study, such as surgery or use of harmful medications. After assessing the STL in these patients, we collected semen from another 104 patients, including 54 patients with a BMI >28 kg/m^2^ and 50 age-matched patients with normal BMIs to assess the impact of BMI on oxidative stress, mitochondrial activity and DNA fragmentation rate in the semen. This study was approved by the Ethics Committee of the First Affiliated Hospital of Zhengzhou University, and informed consent about the usage of data and semen was obtained from all participants. Finally, all the methods used in this study were carried out in accordance with the approved guidelines.

### IVF procedure

The semen analysis was carried out according to the WHO protocol (2010) on the day that the oocytes were retrieved (Day 0). Insemination was conducted by placing cumulus–oocyte complexes (COCs) together with sperm selected via density gradient centrifugation. The cumulus cells were removed after 4 h. Fertilization was confirmed after 16–18 h of insemination. Each Day 3 embryo was evaluated and given a grade from 1 to 5. High-quality embryos included grade I and grade II embryos, which were defined as having six to eight symmetrical equal size blastomeres with no fragmentation (grade I) or less than 10% fragmentation (grade II).

### Telomere length measurement

The average STL was determined in genomic DNA using a real-time PCR for each patient. The PCR reactions were conducted using the 7500 Real-Time PCR System (Applied Biosystems, USA). The telomeric primers, control gene primers (36B4, single-copy gene) and PCR settings were the same as described before^17^. Each sample was conducted in triplicate, and a standard curve was created by serial dilutions of known amounts of reference DNA for each reaction. The telomere to single (T/S) copy ratio was used to calculate the relative telomere length. Laboratory personnel who were blind to the case-control status and the outcome assessment performed all of the measurements.

### ROS production assay

A standardized protocol was used to perform a photometric nitro blue tetrazolium (NBT) assay to measure seminal ROS production via formazan production^53^. After washing the semen in phosphate-buffered saline (PBS) at 300 g for 5 min, the sperm was re-suspended in 200 μl PBS. Then, 100 μl 0.1% NBT (Sigma-Aldrich, USA) was added and incubated at 37 °C for 45 min. The sperm were then washed twice in PBS at 500 g for 5 min. Exactly 60 μl DMSO (Sigma-Aldrich) and 2 M KOH were used to solubilize the intracellular formazan product. The resulting color reaction was measured with a microplate reader at 630 nm (BIO-RAD, Finland). ROS production was expressed as micro-grams of formazan per 10^7^ spermatozoa. A standard curve was obtained from known concentrations of formazan substrate solubilized in DMSO.

### Mitochondrial activity assay

Sperm mitochondrial activity was detected using the method described by Hrudka^54^. Briefly, the semen was diluted 1:3 in PBS with 1 mg/mL 3, 3′-diaminobenzidine (DAB) after semen liquefaction and then transferred to an incubator for 1 h at 37 °C. A 10 μl smear was added to a slide. Then, the air-dried slide was fixed using 10% formaldehyde for 10 min. Exactly 500 sperm were counted for each sample using an Olympus BX51 microscope. The sperm were classified based on the stained length of the midpiece. Classes I to III corresponded to 100%, more than 50%, and less than 50% of the midpiece being stained, respectively. Class IV had no staining in the midpiece.

### DNA fragmentation determination

A sperm chromatin dispersion (SCD) kit was used to detect the DNA fragmentation rate in the sperm according to the manufacturer’s recommendations (BRED, Life Science Technology Inc., Shenzhen, China). Briefly, 60 μl of the diluted raw semen sample was added to an Eppendorf tube and mixed completely with fused agarose heated at 80 °C for 20 min. Exactly 30 μl of the mixture was added to a pre-coated slide, and the slide was cooled at 4 °C for 5 min. The slide was incubated in solution A for 7 min and solution B for 25 min and was then washed in distilled water for 5 min. After dehydration treatment via a series of ethanol concentrations (70–90–100%), the slide was incubated in a mixture of Wright’s solution and PBS for 16 min. The slide was then washed and air-dried. Exactly 500 sperm were counted for each sample using an Olympus BX51 microscope. Sperm nuclei with large- or medium-sized halos were considered non-fragmented, whereas sperm with small nuclei or without halos were considered to contain fragmented DNA.

### Statistical analyses

The demographic data are presented as the means ± SDs for the two groups. The Kolmogorov–Smirnov normality test was used to examine deviations from normal distributions. Student’s *t* test was used to analyze normally distributed data, and Mann-Whitney U test was used to analyze non-parametric data. A chi-square test was used to analyze qualitative data. A general linear regression model was used to assess the relationship between STL and patient BMI. A multivariate logistic regression was used to investigate whether male BMI affects clinical pregnancy after adjusting for female BMI, female age, the number of embryos transferred, basal FSH levels, male age, and sperm count for the overweight BMI group. All statistical analyses were performed using SPSS software Version 16.0 (SPSS Inc., Chicago, IL, USA). A value of *P* < 0.05 was considered significant.

## Additional Information

**How to cite this article**: Yang, Q. *et al.* Effect of paternal overweight or obesity on IVF treatment outcomes and the possible mechanisms involved. *Sci. Rep.* **6**, 29787; doi: 10.1038/srep29787 (2016).

## Supplementary Material

Supplementary Information

## Figures and Tables

**Table 1:** Demographic and IVF cycle characteristics according to male BMI.

Parameter	Male BMI (kg/m^2^)		
	20–25(N = 345)	>28(N = 306)	*P*
Male Age (years)	30.4 ± 4.0	30.5 ± 3.9	0.91
Female Age (years)	29.7 ± 4.9	29.9 ± 3.9	0.48
Female BMI (kg/m^2^)	22.4 ± 3.1	22.3 ± 3.6	0.26
Female Basal FSH (IU/L)	7.4 ± 2.1	6.9 ± 2.5	0.51
Gn duration (days)	11.2 ± 1.9	11.0 ± 1.9	0.13
Gn dosage (IU)	2,034.8 ± 822.6	2,028.5 ± 817.7	0.92
Oocytes retrieved (n)	11.3 ± 5.9	11.2 ± 6.5	0.63
Embryos transferred (n)	2.0 ± 0.3	1.9 ± 0.5	0.32

BMI, body mass index; FSH, follicle stimulating hormone; values are shown as the mean ± standard deviation; Student’s *t* test was used to compare the data between the two groups.

**Table 2:** Basic semen characteristics of the two BMI groups.

Parameter	Male BMI (kg/m^2^)		
	20–25(N = 345)	>28(N = 306)	*P*
Sperm concentration (million/ml)	59.9 ± 54.6	59.5 ± 51.8	0.83
Semen volume (ml)	3.08 ± 1.69	3.79 ± 1.71	<0.01
Total sperm count (million)	234.5 ± 228.3	202.7 ± 155.3	0.04
Sperm motility (%)	46.5 ± 18.9	39.9 ± 19.1	<0.001

Values are displayed as the mean ± standard deviation; Student’s t test was used to compare the data between the two groups.

**Table 3:** Likelihood of clinical pregnancy after IVF, presented as odds ratios (95% confidence intervals).

Male BMI: >28 kg/m^2^(N = 306)	
Unadjusted	0.75 (0.64–0.88)
	*P* < 0.001
Adjusted	0.73 (0.62–0.88)
	*P* < 0.01

A multivariate logistic regression was used to investigate whether male BMI affects clinical pregnancy after adjusting for female BMI, female age, number of embryos transferred, basal FSH levels, male age, and sperm count for the overweight BMI group.
