# Supplementary material for: Effect of paternal overweight or obesity on IVF treatment outcomes and the possible mechanisms involved
Source: Sci Rep. 2016 Jul 14;6:29787. doi: 10.1038/srep29787 (PMC4944201; doi:10.1038/srep29787)
Supplement: Supplementary Information [file srep29787-s1.pdf]

Effect of paternal overweight or obesity on IVF treatment outcomes and the possible mechanisms involved.

Qingling Yang△[1], Feifei Zhao△[1], Linli Hu△[1], Nan Zhang[1], Rui Bai[1], Guidong Yao[1], Yingpu Sun[*1]

Reproductive Medical Center, First Affiliated Hospital of Zhengzhou University, China

△These authors contributed equally to this work.

Running title: **Effect of paternal overweight or obesity on IVF treatment outcomes**

Disclosure summary: The authors have nothing to disclose.

Corresponding author and person to whom reprint requests should be addressed:

Yingpu Sun, MD, PHD.

Center for Reproductive Medicine, The First Affiliated Hospital of Zhengzhou University

40, Daxue Road, Zhengzhou, Henan, China, 450052.

Tel: 86-371-66913635; Email: syp2008@vip.sina.com

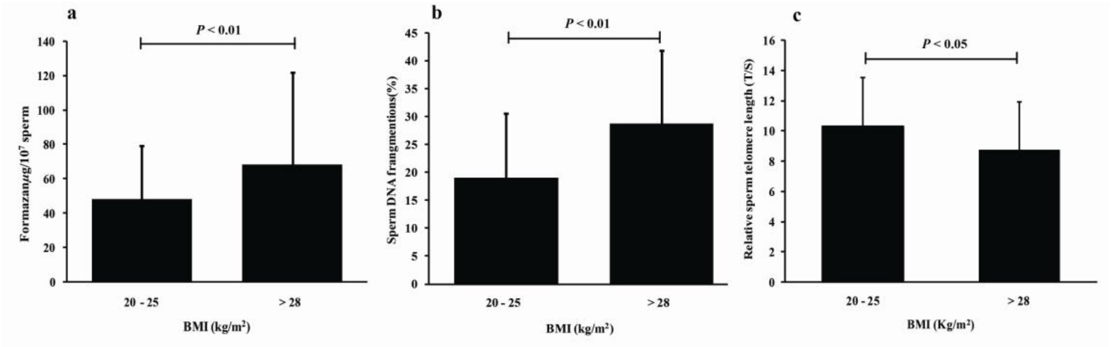

Supplementary Figure 1. ROS production, sperm DNA frangmentation rate and STL in the two groups. a. Comparison of mean ROS production in men with normal BMIs (20-25 kg/m^2; N=50) and those with overweight BMIs (>28 kg/m^2; N=54)(Mann-Whitney U test; bars represent standard deviation). b. Comparison of mean sperm DNA fragmentation rate among men with normal BMIs (20-25 kg/m^2, N=50) and those with overweight BMIs (>28 kg/m^2; N=54) (Student's *t* test; bars represent standard deviation). c. Comparison of relative STL (T/S ratio) in men with normal BMIs (20-25 kg/m^2; N=50) and overweight BMIs (>28 kg/m^2; N=306) (Student's *t* test; bars represent standard deviation).

Supplementary Table 1. Mitochondrial activity between the two BMI groups.

Class	Male BMI (kg/m2)		*P*
	20-25(N=345)	>28(N=306)	
DAB Class I	4.1±2.4	3.5±2.5	0.07
DAB Class II	76.5±6.5	73.8±6.5	0.03
DAB Class III	14.2±4.1	11.8±4.1	0.04
DAB Class IV	5.2±4.1	10.9±4.0	<0.01

Values are shown as the mean±standard deviation; Student's *t* test was used to compare the data between two groups.
